# Adenolipoma of the Breast: A Case Report

**DOI:** 10.31729/jnma.6925

**Published:** 2021-11-30

**Authors:** Samriddhi Karki, Agya Shrestha, Bipin Shrestha

**Affiliations:** 1 Department of Pathology, Nepal Police Hospital, Kathmandu, Nepal; 2 Department of Surgery, Nepal Police Hospital, Kathmandu, Nepal

**Keywords:** *adenolipoma*, *breast*, *case report*, *hamartoma*

## Abstract

Adenolipoma of the breast is a rare tumor classified as a hamartomatous lesion. It is a well-circumscribed lesion composed of adipocytes and other breast tissues. The characteristic feature is a well-circumscribed mass containing radiolucent fat admixed with dense fibrous connective tissue surrounded by a thin radiopaque pseudo capsule. Microscopically, there is a mixture of ducts and lobules with adipose tissue. Ductal hyperplasia, adenosis, calcification, and apocrine metaplasia may occur within the hamartoma. These are rarely associated with malignancies and excision is considered curative. If these lesions are not detected clinically or radiologically, these remain unrecognized. Awareness of this poorly recognized benign entity would help avoid an incorrect diagnosis and unnecessary intervention. Here we present a case of a 35-year-old female diagnosed histologically as adenolipoma of the breast.

## INTRODUCTION

Adenolipoma of the breast is a rare benign lesion that accounts for 0.1-0.7% of all benign breast lesions.^1^ It is often discovered incidentally during mammography. Clinical findings resemble fibroadenoma and are characterized by a painless, smooth mobile breast lump that may or may not be palpable and slowly enlarge in size.^2^ Histopathological finding includes mass composed of ducts and glandular structures randomly embedded in mature fatty tissue.^3^ Adenolipoma should be differentiated from other lesions of the breast to avoid an incorrect diagnosis and unnecessary intervention. Here we report a case of a 35-year-old female diagnosed histologically as adenolipoma of the breast.

## CASE REPORT

A 35-year-old female presented to the surgical outpatient department (OPD) with the complaint of a right breast lump for three years. There was no history of nipple discharge, fever, or weight loss. Other medical and family histories were insignificant.

**Figure 1:**
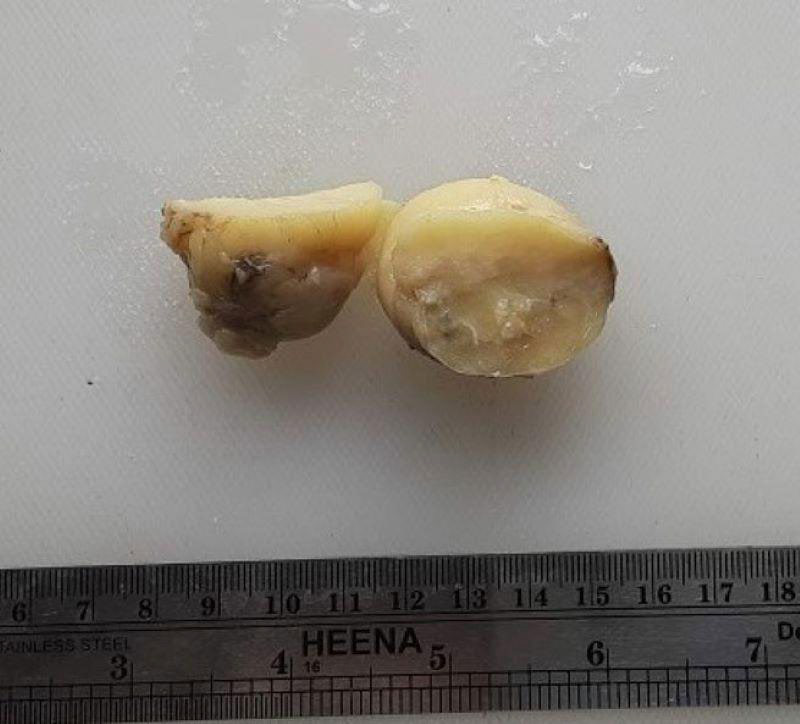
Well circumscribed encapsulated mass with yellowish cut surface and focal grayish-white areas.

Physical examination revealed a single, non-tender, regular, mobile mass in the upper outer quadrant of the breast. The overlying skin was normal. Nipple discharge and retraction were not detected. There was no axillary lymphadenopathy. The left breast was normal. Ultrasonography showed a well-circumscribed, heterogeneous, hypoechoic mass in the right breast measuring approximately 4.7x3.9x2.2cm at 2 o'clock peri areolar region with a likely differential diagnosis of lipoma and fibroadenoma. Fine needle aspiration (FNAC) showed the presence of benign ductal epithelial cells admixed with myoepithelial cells arranged in sheets and clusters. The background showed plenty of fatty fragments and was reported as benign breast disease. An excisional biopsy was performed on the right breast. Histopathological examination was done. Macroscopically, a 5x4x2cm, yellowish, oval, encapsulated mass was found. Cut surface showed yellowish areas with focal grayish-white areas (Figure 1).

**Figure 2:**
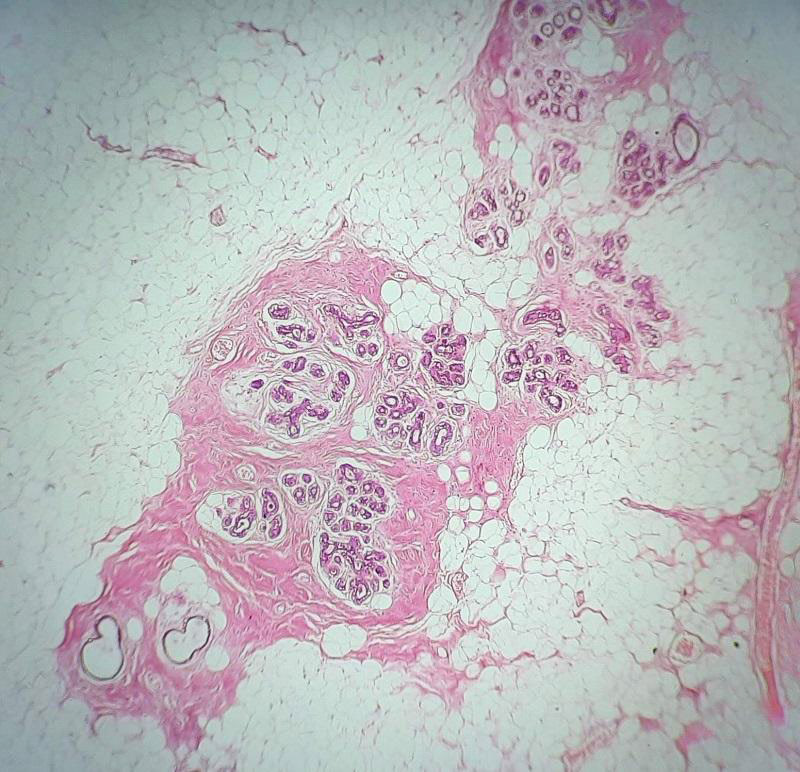
Tumor tissue is composed of mature adipose tissue along with breast ducts and lobules surrounded by fibro collagenous tissue (H&E x400).

Microscopically, the tumor was surrounded by a fibrous pseudo capsule and consisted of mature adipocytes admixed with areas of breast ducts and lobules which are surrounded by fibro collagenous tissue (Figure 2).

No proliferative changes in lobules and ducts were identified within the lesion. Based on these histological findings, a diagnosis of adenolipoma was made. The patient was followed up after 3 months with an ultrasound report which showed no recurrence.

## DISCUSSION

Hamartoma refers to the presence of a disorganized mixture of components that are endogenous to a particular site. Breast hamartomas are rare benign lesions that may recur. These are composed of a random mixture of glandular epithelial, fibrous and fatty tissue. The exact pathogenesis of breast hamartoma is still unknown. However, it is believed to be a result of a development abnormality rather than a true neoplastic process.^2^ Hamartomas express estrogen and progesterone receptors and exhibit an increased proliferative activity.^4^

Adenolipoma is the most common variant of breast hamartomas, suggested first in 1945 by Spalding.^3, 4^ They are frequently seen in premenopausal women, mostly in their forties due to involution of the breast tissue which makes these lesions more apparent with asymmetrical enlargement.^2,5^ As in our case, they are typically found on the outer quadrant of the breast with a slight tendency towards the right.^5^ It can present with a clinically palpable mass or can be incidentally diagnosed during mammography in nearly 60% of cases.^2^ Mammographic finding varies depending upon the presence of a degree of each of its constituents and is classically identified as well-circumscribed, round to oval masses containing fat and soft tissue densities with a thin, radiopaque pseudo capsule.^2,6^ Mass is usually well defined, soft, painless and mobile. The size of the lesions ranges from 1 to 20 cm in diameter in reported cases.^2, 3^

Grossly, mass is well-demarcated, occasionally lobulated with smooth contours and often rubbery greyish white to yellow cut surface, resembling fibroadenoma or lipoma.^7^ Lesions with abundant fat resemble lipomas as in our case.

Microscopically, the mass is pseudo encapsulated and composed of mammary ducts, lobules, fibrous tissue, and adipose tissue in varying proportions. Additional changes like pseudo angiomatous hyperplasia, fibrocystic changes, adenosis, and prominent smooth muscle proliferation may also be seen.^2, 5^

Although hamartomas are rarely associated with malignancies, multiple hamartomas that have been associated with certain genetic abnormalities like Cowden syndrome, which is an autosomal dominant disorder mainly arising from the germline mutation of the PTEN gene, are linked with increased risk of breast cancer and development of hamartomas in other parts of the body.^4, 5 7^

Surgical excision is the treatment of choice.^5^ Postoperatively, patients are suggested to follow up with mammography and ultrasound every 6 months for 1-2 years to monitor for any recurrence.^2^ Due to its rare association with malignancies and nonspecific clinicoradiological findings, surgical excision with histopathological examination is necessary for a definite diagnosis.
